# Mr. Saumarez on Generation and the Principle of Life

**Published:** 1799-11

**Authors:** 


### *Mr.* SAUMAREZ *on Generation and the Principle of Life.*

[ Continued from p. 242—247 of our laſt Number. ]

THE fact is proved by the pollen of vegetables, by the ſemen and ova of fiſh, and of the amphibia which I already ſtated. Being ſolicitous to ſee what change the ovaria underwent by the power of œſtrum alone, I took a female rabbit that had the œſtrum upon her, and had her fed upon oats, beans, cellery, and other kinds of food which the rabbit-keeper told me had the ſtrongeſt tendency to increaſe that ſtate. I had her placed before a buck; they were allowed to careſs each other whilſt abſolute union was prevented; I purſued this plan for a week, and at the time that the œſtrum was at its higheſt pitch, ſhe was killed. On examining the different organs ſubſervient to the proceſs of generation, I found them very different from what they are in a common ſtate.—The external membrane by which the vagina is lined was ſwelled and diſtended, and had acquired a black mulberry colour; on examining the uterus, I found its colour had undergone an equal alteration; it was of a purple hue, evidently ariſing from a præternatural quantity of blood that had been determined upon it. There was a large veſſel running up the middle of both fallopian tubes, enlarged to a conſiderable ſize, and completely diſtended with blood. The tubes before their termination at the fimbriæ were torquated and diſtorted in an extraordinary manner, having alſo a ſtrong periſtaltic motion—after running a ſhort way above the ovaria, they bent downwards, terminating by a fimbriated expanſion above the ovaria, a conſiderable portion of which they involved and encloſed. The ovaria appeared to have undergone a conſiderable degree of alteration alſo; the ova which the ovaria contained were ſwelled, and evidently more diſtinct than is uſual, reſembling in ſome degree the ſeed reſident in the pericarpium of a ripe grape. Although it appeared very clear that ſome action had taken place in theſe parts, there was nothing like a ſeparation from the capſule, as we obſerve in the ovarium of the hen, from the effect of œſtrum alone, (without ſexual union.) Œſtrum alone therefore produces an evolution of the ova to a limited degree; it however appears from ſome experiments that were made by Mr. CRUIKSHANK and Dr. HAIGHTON, where one of the fallopian tubes of a rabbit had been divided and obliterated, and the other left perfect and entire, and ſexual union allowed, that the encreaſed excitement which the female had undergone was ſufficient to evolve the ova completely, and ſeparate them from the ovaria

Although there were ova feparated from the ovarium in the mutilated as in the perfect fide, there was this grand and ftriking difference between both. In the perfect fide where the femen could have accefs to the ovarium, there were fœtufes found as ufual; on the mutilated fide quite otherwife, there was not the leaft trace of a fingle fœtus to be difcovered. Although there was not a fingle fœtus to be difcovered in the mutilated fide, both ovaria difplayed the fame appearance; the ova which the ovaria contained, I fay, became equally evolved, the external tunic in both had burft, and feveral of the ova in both were difcharged. The veficles from whence the ova were difcharged were confequently left hollow, their parieties or fides gradually thickened; and thefe thickened calyces conftitute what Anatomifts have called *corpora lutea*. The exiftence, therefore, of *corpora lutea* is a proof that the fexual act has been fo far perfect as to produce this action within the veficles; and that although it can take place without the application of femen to the ovaria, fœcundation is abfolutely prevented without its palpable application. The conclufion therefore preffes itfelf upon the mind with force irrefiftible, that, *the exiftence of corpora lutea is not the true and infallible teft of animal impregnation.* It is not more the teft of animal impregnation, than when we behold unfœcundated eggs expelled from the female of oviparous animals in general; in them, the feparated part of the membrane is left jagged, and is exactly analagous to the corpus luteum of the higher order of animals.

If male and female fifh, of the fame fpecies, are feparated from each other, although the fpawn will be fhed, fœcundation will be effectually prevented. If birds that ufually copulate together, are cooped up feparate, although the eggs will evolve and be depofited, they will be totally different from what we find when the fexual act has been accomplifhed: in the one cafe, they become putrid without being prolific; in the other, they become prolific without being putrid. Although the fhell of a perriwinkle and the hide of a buffalo are different in their ftructure, they are deftined to the fame ufe, ferving to protect from the operation of external caufes the internal organization of the animal to which it is fubfervient. We have no difficulty in admitting, that the cornuated uterus of a rabbit is deftined for the fame purpofe as the oval uterus of the human fpecies, the oviduct of a hen for the fame purpofe as the fallopian tube of a woman; muft it not then be allowed, that the ovaria of the one are in nature the fame as the ovaria of the other? and that fince fœcundation cannot take place without the application of femen to the ovaria, although *corpora lutea* do exift, that the prefence of thefe is not the teft by which we are to judge that fœcundation has actually tually taken place? The only certain teſt we can have that animal fœcundation has taken place, is by the actual exiſtence of one or more fœtuſes. It is not an effect produced from the energy of a power reſident in one ſyſtem or in one ſex, but in two ſyſtems of different ſexes; not in the male or female individually, but from the united action of both male and female together.

From the various facts I have ſtated, I think we are warranted in concluding,

1ſt. That the act of ſexual intercourſe is the immediate cauſe, by the power of which the ſeveral organs in the male and female are made to undergo their ſeparate although correſpondent changes.

2d. In the male the ſpecific power of the teſtes is excited, and ſemen in conſequence produced; which ſemen is the immediate agent that contains the characteriſtic properties of the maſculine ſyſtem, and is conveyed from the vagina of the female through the uterus, and received by the fallopian tubes. In the female, the increaſed vaſcularity not only of the vagina but of the uterus and tubes, proves the capacity theſe parts poſſeſs of ſympathizing with the ſexual organs of the male.

3d. That the fallopian tubes conſtitute the media of communication to convey the ſemen from the uterus to the ovaria, which they do by means of the periſtaltic power which theſe tubes ſo eminently poſſeſs; that in proportion as the evolution which the ova ſuſtain (in the veſicles of the ovaria) a correſpondent change takes place in the fimbriæ; that the fimbriæ progreſſively graſp the ovaria, and immediately apply the ſemen to the ova; that by the union of both, fœcundation takes place, and which conſtitutes the proximate cauſe of animal impregnation.

In order to put ſo difficult a ſubject in as clear a point of view as I am capable, I ſhall ſtate it in different words, viz. the different changes which the ſeveral parts in the male and female undergo, all tend to one end, namely, the immediate contact of the fimbriated extremity of the fallopian tubes to the ſurface of the ovaria; that an union might take place between the fluid which the fimbriæ convey, and the ova (of a mucus-like appearance) which the veſicles diſcharge; that it is the ſemen which communicates the characteriſtic properties of the male; and the fallopian tubes, the medium by which it is conveyed, in the ſame manner as the fluid which the veſicles diſcharge, contain and convey the characteriſtic properties of the female;—that the fimbriated extremity of the fallopian tubes, is the immediate ſeat where this effect takes place; that the union of both conſtitutes *conception*, or the immediate reception of of a *living principle*, in which the ſource and power of action eſſentially reſides, and which participates the nature of both parents, by the combined action of whom it was produced.*

It is not the farinaceous matter of which a ſeed is compoſed, the animal gluten with which an egg is filled, or the atom of mucus in which the primordia of the embryo are contained, which conſtitutes the power by the energy of which organization is elaborated and action ultimately produced; each of theſe bodies is a chaos in all its parts, a *rudis indigeſtaque moles*; the mere matter of which they are compoſed, when deprived of this living principle, undergoes the uſual changes of decompoſition and decay, by the proceſſes of putrefaction and fermentation, and yields nearly the ſame materials by chemical analyſis.

It is the living principle which the ſeed of vegetables and the fœcundated ova of animals contains, that conſtitutes the architect by which the machine is erected; it is the baſe on which the whole ſtands, it is the bond of its elementary parts, the cement that unites them in one whole;—it is the efficient and primary cauſe from whence the individuality of every ſyſtem ariſes, and in which the form it aſſumes eſſentially reſides;—it conſtitutes the power by which the human ſpecies differs from the brute, the brute from the vegetable, the vegetable itſelf from formleſs and inanimate matter;—it is the power by which formleſs and inanimate matter is converted into organs living and active, ſo that the various ſpecies of food which the vital power receives, are nothing more than the raw materials applied to it;—it is the manufacturer that converts theſe materials, without power, or intelligence into different ſyſtems—through which the acorn becomes evolved into an oak, the infant foliage expanded into leaves, and the final cauſe of vegetable exiſtence attained;—it is by virtue of a power in eſſence the ſame, though in character different, that the embryo of animals becomes evolved out of matter in kind the ſame, and models a ſyſtem conſiſting of organs and of fluids in

* That fœcundation takes place either at the extremity of the fallopian tube, or in the very calyx itſelf, which is formed by the action of the veſicle after the ſexual act, is not only very probable, from the appearance which the parts diſplay, but the probability is greatly increaſed from the adventitious circumſtances which ſometimes happen, when a fœtus is found reſident either in the ovarium, or attached to the fimbriæ, or lodged within the body of the tube itſelf; or, what perhaps leſs rarely happens, when the embryo drops from either of thoſe ſituations, and becomes attached to ſome part of the abdominal cavity: Theſe are called extra-uterine caſes in general, each caſe receiving the particular denomination from the particular part in which it is found, as, abdominal, ovarial, fimbrial, and fallopial.

in their kind and operation totally different; and, finally, it is through the participation of this living power, which the organs and fluids have received, that they become the inftruments or means by which it accomplifhes the final caufe of its exiftence:—*Life* may therefore be defined—*the principle (i. e. the efficient and primary caufe) by the energy of which various fpecies of matter are converted to one kind, under one fyftem, fo that the matter thus converted poffeffes the power of refifting the operation of external caufes, and of preferving itfelf from putrefaction and decay.* It is to this power, I fay, by the energy of which every living fyftem is protected and preferved from decompofition and decay, and by which the different fubftances it receives are affimilated and changed, that I attach the idea of life.—The Vis Medicatrix Naturæ of STAHL, the Vis Vitæ of HALLER, the Nifus Formativus of BLUMENBACH, the Living Principle of Mr. HUNTER, the Excitability of Dr. BROWN, and, finally, "Form," by that excellent philofopher Mr. HARRIS:—The Principle of Life, therefore, as a caufe, may be contemplated in the abftract, as feparate and diftinct from the matter into which it is received, and through which its actions are produced; it is by the evolution of the living principle which animated beings poffefs, from a ftate of *dormant capacity* into energy and action, that they are capable of converting to their own nature, the various fubftances on which they feed, and of making them affume the organization and form of the fyftem to which they are applied. Bread of the fame precife quality, cut from the fame loaf, or water drawn from the fame brook, given to a man and to a dog, after having been digefted by the ftomach of both, will contribute to the particular organization of each refpective fyftem. We behold a multitude of vegetables placed in the fame medium, nourifhed and fed by water and air, in kind the fame, and yet affuming an organization and form totally different.

If the power of organization and of life therefore refided in the food, every vegetable and every animal that fed upon the fame materials would be fafhioned and modelled alike; for in all chemical changes the fame caufe uniformly produces the fame effect.—If it depended on a chemical caufe, the changes which the food fuftained would be regular and conftant, the chyle produced, inftead of being the fame, would be generally different; it would vary in its properties according to the peculiar nature of the fubftances out of which it was formed; and, finally, if it depended on the mutual action of different parts of the food upon each other, independant of the digeftive power of the organ itfelf, the change it fuftained, like other chemical changes, would be conftant and and definite, and not liable to the remiſſion we witneſs during the proceſs of digeſtion;—it is therefore lawful, and we are from neceſſity, led to conclude, that the commutation food obtains in the living ſyſtem, is a vital and not a chemical act; and that the efficient cauſe of this commutation does not ariſe from any active property which the food contains, but is owing to the vital power of the ſyſtem in which it is received, and by which the new arrangement of its parts is formed; it is with a view of deſtroying theſe ſenſible qualities which different living ſyſtems receive, that their aſſimilating organs are eſſentially deſigned; they are deſigned to reduce ſubſtances of different kinds to one, that this one ſubſtance may be in harmony with the ſyſtem, that it may be fitted for being acted upon, and converted by the ſpecific power of different organs into various ſhapes; the aptitude therefore of the matter which every living ſyſtem receives, can only ariſe out of its weakneſs or total privation.—It is in this deſtitute ſtate that we ſay matter is imbecile and inert, a mere *tabula raſa*, that has the aptitude of being acted upon without the power of reſiſting action; that has the power of being changed without the power of changing; of being modelled without the power of modelling, &c. it is thus that we can appreciate the dire ignorance of thoſe materialiſts, who ſuppoſe that matter can convert *itſelf* into different organs, in fabric moſt delicate, in action moſt extenſive, in form moſt diverſified; that by the congregation of theſe organs a whole ſyſtem is conſtituted; that the reſult of this organization is life, and out of this organized life, action and motion are produced, ſo that matter is the efficient cauſe, and life the effect only.

Equally abſurd is the opinion of thoſe pure defecated philoſophers who ſuppoſe that the oxygenous matter which vegetables in the day are conſtantly diſcharging from the whole external ſurface of their foliage as urinous and dead, conſtitutes the principle of life, in which all power eſſentially reſides—the immediate and proximate cauſe of irritability in man!

With ſtill greater reaſon are we to deplore the Brunonian ſyſtem, which proclaims life to be a *forced*, not an original ſtate—that makes life the effect of action, inſtead of action the effect of life—that makes life to come out of the body inſtead of reſiding within it!—*that makes this action, or excitement, in which the true cauſe of life conſiſts, the effect of the exciting powers acting on the excitability!* &c. &c.

